# Scanxiety Conversations on Twitter: Observational Study

**DOI:** 10.2196/43609

**Published:** 2023-04-19

**Authors:** Kim Tam Bui, Zoe Li, Haryana M Dhillon, Belinda E Kiely, Prunella Blinman

**Affiliations:** 1 Medical Oncology Concord Cancer Centre Concord Australia; 2 Sydney Medical School University of Sydney Camperdown Australia; 3 Psycho-Oncology Cooperative Research Group School of Psychology University of Sydney Camperdown Australia; 4 Centre for Medical Psychology and Evidence-based Decision-making University of Sydney Camperdown Australia

**Keywords:** anxiety, cancer, medical imaging, oncology, psycho-oncology, social media, twitter, tweet, scanxiety, mental health, sentiment analysis, oncology, thematic analysis, screen time, scan, hyperawareness, radiology

## Abstract

**Background:**

Scan-associated anxiety (or “scanxiety”) is commonly experienced by people having cancer-related scans. Social media platforms such as Twitter provide a novel source of data for observational research.

**Objective:**

We aimed to identify posts on Twitter (or “tweets”) related to scanxiety, describe the volume and content of these tweets, and describe the demographics of users posting about scanxiety.

**Methods:**

We manually searched for “scanxiety” and associated keywords in cancer-related, publicly available, English-language tweets posted between January 2018 and December 2020. We defined “conversations” as a primary tweet (the first tweet about scanxiety) and subsequent tweets (interactions stemming from the primary tweet). User demographics and the volume of primary tweets were assessed. Conversations underwent inductive thematic and content analysis.

**Results:**

A total of 2031 unique Twitter users initiated a conversation about scanxiety from cancer-related scans. Most were patients (n=1306, 64%), female (n=1343, 66%), from North America (n=1130, 56%), and had breast cancer (449/1306, 34%). There were 3623 Twitter conversations, with a mean of 101 per month (range 40-180). Five themes were identified. The first theme was experiences of scanxiety, identified in 60% (2184/3623) of primary tweets, which captured the personal account of scanxiety by patients or their support person. Scanxiety was often described with negative adjectives or similes, despite being experienced differently by users. Scanxiety had psychological, physical, and functional impacts. Contributing factors to scanxiety included the presence and duration of uncertainty, which was exacerbated during the COVID-19 pandemic. The second theme (643/3623, 18%) was the acknowledgment of scanxiety, where users summarized or labeled an experience as scanxiety without providing emotive clarification, and advocacy of scanxiety, where users raised awareness of scanxiety without describing personal experiences. The third theme was messages of support (427/3623, 12%), where users expressed well wishes and encouraged positivity for people experiencing scanxiety. The fourth theme was strategies to reduce scanxiety (319/3623, 9%), which included general and specific strategies for patients and strategies that required improvements in clinical practice by clinicians or health care systems. The final theme was research about scanxiety (50/3623, 1%), which included tweets about the epidemiology, impact, and contributing factors of scanxiety as well as novel strategies to reduce scanxiety.

**Conclusions:**

Scanxiety was often a negative experience described by patients having cancer-related scans. Social media platforms like Twitter enable individuals to share their experiences and offer support while providing researchers with unique data to improve their understanding of a problem. Acknowledging scanxiety as a term and increasing awareness of scanxiety is an important first step in reducing scanxiety. Research is needed to guide evidence-based approaches to reduce scanxiety, though some low-cost, low-resource practical strategies identified in this study could be rapidly introduced into clinical care.

## Introduction

“Scanxiety,” or scan-associated anxiety, was a term first coined by a patient writing for *Time* magazine to describe the distress before, during, or after a scan [1]. Scans are often routine in cancer care [2] regardless of cancer type or stage. They are performed for screening, diagnosis, surveillance, and monitoring of cancer and may occur on a regular schedule or in response to new symptoms, signs, or other investigation results. Global cancer incidence has increased over time, with over 20 million new cancers diagnosed annually [3,4]. Cancer survival has also increased over time secondary to improved detection of cancer and the efficacy of anticancer treatments [5,6]. Understanding the impact of scans on patient experiences is valuable, especially as improved cancer survival means more people are living with cancer and more scans are being performed over the course of the cancer journey of a patient [7].

Quantitative research on scanxiety was summarized by a scoping review in people having cancer-related scans [8]. The number of studies (n=57) indicated scanxiety was a clinically important problem, though the range of scanxiety prevalence (between 0% and 83%) was affected by methodological heterogeneity in cancer types, scan modality, and the tools and timing of scanxiety measurement [8].

Meanwhile, qualitative research on scanxiety has focused on physical factors [2,9-14]. Participants described discomfort around positioning, claustrophobia, noise, duration, temperature, cannulation, or contrast. Scanxiety was exacerbated by unfamiliarity with scans and by unempathetic or uncommunicative radiology staff [2,9-14]. A minority of studies acknowledged that scanxiety can occur while waiting for scan results [9,10,12,13]. These studies used traditional research methods such as interviews and focus groups and were limited by selection bias and the difficulty of generalizing results. They had modest sample sizes (4 recruited under 20 participants [2,10-12]), recruited participants with an extended time since their cancer diagnosis (1 with a median of nearly 6 years [10]), or recruited participants from uniform demographic groups [13].

A novel approach to data collection to supplement traditional methods is through web-based cancer communities, which can provide important perspectives on health issues, inform research, be used for health interventions, and enable the sharing or dissemination of information and research findings [15-17]. These communities can be hosted on social media platforms like Twitter, which had over 300 million global users at the time of this study’s inception [18]. On Twitter, users post real-time messages limited to 280 characters (“tweets”) [19], with the potential for users to provide a unique perspective on scan experiences and scanxiety in people having cancer-related scans. The transient phenomenon of scanxiety, which often mirrors the periodic nature of cancer-related scans, may be optimally captured on Twitter given the accessibility of Twitter on internet-enabled mobile and computer devices as well as the ease of posting contemporaneous tweets.

This study aimed to identify and describe Twitter activity about scanxiety by determining the demographics of users who posted about it, and the volume and content of these tweets.

## Methods

### Overview

We conducted a manual search of Twitter to identify relevant tweets published between January 2018 and December 2020. We used the following search terms: “scanxiety,” “scananxiety,” “scan anxiety,” “scan-anxiety,” “scan-related anxiety,” and “scan-associated anxiety.” Tweets were grouped into “conversations,” consisting of primary and subsequent tweets. Primary tweets were the first tweets about scanxiety in a conversation. Subsequent tweets were comments or retweets stemming from the primary tweets.

The search strategy output within their web browsers was independently reviewed by 2 authors (KTB and ZL). Included were primary tweets that were publicly available, in English, and related to cancer. Duplicate tweets or those clearly not related to cancer were excluded. Included tweets were extracted into an Excel (Microsoft Corp) document in chronological order. Uncertainty about whether a tweet met eligibility criteria prompted an additional discussion between the 2 authors and a review of user profiles and other tweets by the same author to provide context about whether the tweets referred to a cancer-related scan. All authors were available for additional review if a consensus was not reached, but this was not required.

Relevant data were manually extracted into a standardized electronic data collection form in the Excel document. Data about the tweet itself was extracted, including the date of the tweet, its classification as a primary or subsequent tweet, the content of the tweet (extracted verbatim), the search term used to identify the tweet, and the use of hyperlinks, media, or emojis within the tweet. Demographic data about users who posted a primary tweet were extracted from the user profile on Twitter, including primary role (patient, family or friend, clinician, organization, researcher, advocate), cancer type, gender (male, female, not specified, not applicable), and location. Gender was not applicable for users representing a group or organization.

### Objectives and Assessments

We had 3 main objectives. The first was to describe the population who posted primary tweets about scanxiety.

The second objective was to determine the volume of conversations about scanxiety by quantifying the total number of conversations over the prespecified time period. Changes in the number of conversations over time were graphed.

The final objective was to explore content about scanxiety. Conversations underwent inductive thematic analysis through simultaneous data collection and analysis, allowing familiarization with the data and coding of the data into themes. Themes were iteratively reviewed and updated through concept mapping and active discussion among all authors, which included medical oncologists and a behavioral scientist. Theme names were chosen to be in plain language, unique to one another, and purposefully neutral to reduce interpretation bias. Once final themes were determined, all primary tweets were rereviewed by 1 author (KTB), who assigned a predominant theme to each tweet. All authors were available to resolve coding uncertainties, but this was not required. Content analysis was conducted to capture the number of primary tweets using hyperlinks, media, and emojis. Words and phrases used to describe scanxiety were extracted from the data set by manual review of the data collection form by the authors and then compiled using a digital word art creator [20]. Greater text size reflected both the manual selection of words and phrases with greater emotional impact as well as automatic adjustments made by the program’s inbuilt algorithm.

### Ethics Approval

This study was approved by the University of Sydney Human Research Ethics Committee (2020/868). Although the research was performed on publicly available Twitter content, a precautionary waiver of consent was granted.

## Results

### User Demographics

There were 2031 unique Twitter users who initiated conversations about scanxiety (Table 1). Most were patients (n=1306, 64%), female (n=1343, 66%), and from North America (n=1130, 56%). Patients most commonly had breast (449/1306, 34%), bowel (150/1306, 11%), or brain (102/1306, 8%) cancer.

**Table 1:** Demographics of people who initiated a conversation about scanxiety (N=2031).

		Participants, n (%)
**Role**		
	Patients	1306 (64)
	Organizations	254 (13)
	Family or friends	251 (12)
	Clinicians	128 (6)
	Advocates	40 (2)
	Researchers	16 (1)
	>1 role	19 (1)
	Unclear	36 (2)
**Gender**		
	Female	1343 (66)
	Male	393 (19)
	Not specified	32 (2)
	Not applicable	263 (13)
**Location**		
	North America	1130 (56)
	United Kingdom	674 (33)
	Australasia	76 (4)
	Other	66 (4)
	Unclear	85 (4)
**Most common cancer types**		
	Breast	514 (25)
	Brain	206 (10)
	Bowel	170 (8)
	Hematological	102 (5)
	Lung	88 (4)
Verified account		34 (2)

### Volume of Tweets

**Figure 1:**
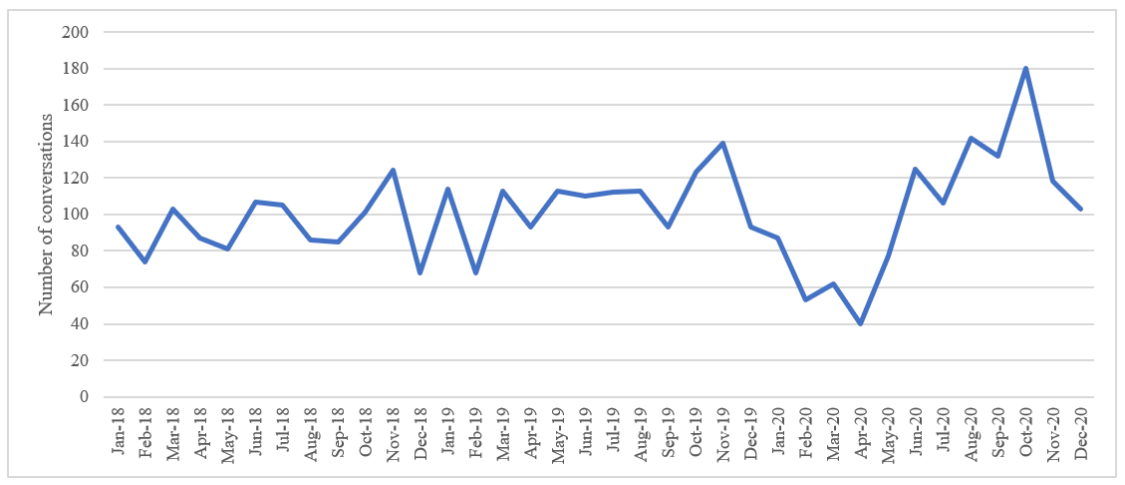
The number of Twitter conversations about scanxiety by month.

There were 3623 Twitter conversations about scanxiety over the 3 years, with 56% (n=2031) initiated by a unique user. Most included the search term “scanxiety” (n=3312, 91%; Multimedia Appendix 1). There was a mean of 101 tweets per month (range 40-180; Figure 1).

### Content of Tweets

#### Overview

Five themes identified were experiences of scanxiety (2184/3623, 60%), acknowledgment of and advocacy for scanxiety (643/3623, 18%), messages of support relating to scanxiety (427/3623, 12%), strategies to reduce scanxiety (319/3623, 9%), and research about scanxiety (50/3623, 1%).

Primary tweets contained hyperlinks, media, or emojis in 21% (746/3623), 20% (709/3623), and 21% (744/3623), respectively. Twitter users included hyperlinks to their personal blogs (414/746, 55%) or strategies to reduce scanxiety (153/746, 21%). They included photos of themselves (206/709, 29%) or photos related to their scanning experience (90/709, 13%). They used a range of emojis to express a positive, negative, or supportive sentiment or to provide a visual depiction of their words (Multimedia Appendix 2).

#### Theme 1: Experiences of Scanxiety

Experiences of scanxiety included a personal account of it by patients or their support person. Scanxiety was often described with negative adjectives or similes (Multimedia Appendix 3).

Scanxiety was experienced differently by users. Scanxiety was often episodic, where users lived “scan to scan,” held their breath “for 72 hours every 3 months,” or felt that “every 3 months, cancer makes me feel like a death row prisoner hoping for a stay of execution.” Others felt they were “stuck in constant scanxiety” with scans every 6 weeks. Scanxiety could get “worse every time,” be never-ending (“86 times and I still get scanxiety”), dissipate over time (“I think I’ve finally mastered scanxiety”), or occur for the first time a few years after diagnosis. Around a single scan, users sometimes felt scanxiety for days (“the last month has been lost to scanxiety”) or would notice a peak (“today is results day and our nerves are shattered”). It could occur as a “low simmering bubble” or like “living on a knife edge.” Users expressed the presence of scanxiety through countdowns to their scan results (“It is only 96 hours, 47 minutes, and 34 seconds”). Some users reported minimal scanxiety, believing that “no amount of overthinking will change the result.”

There were psychological, physical, and functional impacts of scanxiety. Users catastrophized (“I plan my funeral during scans”), were hyperaware of symptoms (“the moment I receive my appointment letter, every twine, pain, or ache is suddenly attributed to my cancer”), ruminated (“I wish my brain had an off switch”), found it “hard to stay positive,” or felt mentally frail (“I am barely hold it together”). The psychological burden was sometimes added to “normal” anxiety levels, while others reported that scanxiety occurred despite their usual optimism.

A common physical symptom of scanxiety was insomnia, where users were unable to sleep, woke early or during the night, or had shortened sleep duration. Users reported tremors, anorexia, abdominal pain, nausea, lethargy, and irritability. Some had poor concentration (“my mind is miles away from where I need it to be”). Some were tense and could not “remember how to relax.” Some experienced panic attacks, teeth-grinding, nail-biting (“we’ve entered the ‘rip off all my cuticles’ phase of scanxiety”), and tearfulness.

Functionally, users noted decreased productivity (“I would show you how I handled scanxiety, but no one needs to see the sink filled with dirty dishes that I didn’t do”), stasis in their lives (“I will not be making plans until I know whether I get to have my next 3 months as not-cancer months”), antisocial behaviors (“I disappear for a while to deal with my emotions”), or reported health care consequences where they would delay appointments for scan results.

Users also described factors contributing to scanxiety. A recurring factor was the presence and duration of uncertainty (“the worst part” and “a difficult friend”), especially while waiting for scan results. Some waited weeks to months for scan results, lamented delays due to long weekends or holidays, and described helplessness (“all I can do is wait”). Scanxiety occurred despite the likelihood of cancer recurrence or progression. One person stated, “brain says everything points to a good, stable result. My heart and stomach have their doubts.” The duration of uncertainty was extended, and scanxiety was exacerbated, when results were not ready in time or when users were promised a phone call for results that did not eventuate. Users described side effects from scans (“queasy stomach,” “taste of metal,” and “claustrophobia”) or procedural issues (“they can’t find a vein...Feel like a pin cushion”).

The ongoing COVID-19 pandemic also contributed to scanxiety, as it caused scan delays or cancellations. Policies on visitor limits meant patients had scans and received results alone. Users were worried about getting COVID-19 when coming for appointments for scans or results. Some users likened their experiences with scanxiety to the unease, fear, and anxiety people experienced during the pandemic.

#### Theme 2: Acknowledgment of, and Advocacy for, Scanxiety.

Acknowledgments of scanxiety included statements without emotive clarification or when users summarized another person’s experience using the term scanxiety. Users stated: “scanxiety is real,” “scanxiety exists,” or simply “Scanxiety.” Others stated, “the unofficial term is scanxiety” or “we in the cancer community call it scanxiety.” These acknowledgments were often posted by patients as commentary about their own experiences or in response to another patient’s experiences.

Advocacy for scanxiety included tweets that raised awareness about scanxiety without mentioning personal experiences and were mostly posted by patients, their families and friends, and organizations. Users stated that scanxiety was “not spoken about often enough” and advocated for the recognition of the term scanxiety. Tweets included hyperlinks to blogs, news articles, podcasts, or videos about scanxiety, as well as invitations to join discussion groups, webinars, or support groups on the topic.

#### Theme 3: Messages of Support Relating to Scanxiety

Twitter users expressed support for people experiencing scanxiety through well wishes and by encouraging positivity. Messages were often posted by patients or family and friends who were able to empathize with the scanxiety experience. Users provided reassurance to people having scans by stating scanxiety as normal and relatable and by offering assistance (Multimedia Appendix 4).

#### Theme 4: Strategies to Reduce Scanxiety

Users adopted or recommended strategies to reduce scanxiety (Table 2). These involved general or specific strategies for patients or strategies requiring the involvement of health care professionals or systems. Patients posted about strategies they used or wanted, while organizations posted about strategies to offer advice to patients. Advocates were more likely to post about strategies requiring a change in the practices of health care professionals or the processes of health care systems.

**Table 2:** Adopted or recommended strategies to reduce scanxiety.

Category		Examples
**General patient strategies**		
	Distraction	Dietary intake: alcohol, coffee, desserts Exercise: pilates, walking, running, cycling, swimming Socializing with friends, family, and pets Creative outlets: music, art journaling, drawing, writing Entertainment: games, reading, shopping, movies, television Mental engagement: mathematics Productive activities: cleaning, organizing, cooking, making soap
	Relaxation	Physical: yoga, deep breathing, aromatherapy, massage, tai chi, acupuncture Mental: meditation, spa music, mindfulness
	Spiritual practices	Prayer, reading the bible or Buddhist teachings
	Seeking support	Requesting well wishes Sharing experiences with family and friends, on forums, in support groups or digitally Self-education on scanxiety via blogs, websites, workshops, or webinars
	Seeking professional support	Speaking with oncology psychologists or social workers Cognitive behavioral therapy Hypnotherapy
**Specific patient strategies**		
	Psychological approach	Methodological (taking “one day at a time”) Pragmatic (“no amount of overthinking will change the scan result”) Optimistic (“I focus on time I’ve already been given – far more than I could have expected”) Contextualizing by comparing their experiences to others Problem-solving by recognizing and minimizing personal triggers to scanxiety Positive self-talk
	Practical	Booking scan and appointment for results close together Antianxiolytics use Building relationships with radiology staff
**Strategies for health care professionals or health care systems**		
	Patient education	Around: scan procedures, results procedures, presence of scanxiety, strategies to reduce scanxiety
	Clinician education	Around: presence of scanxiety, clinician actions to reduce scanxiety
	Clinician actions (oncologists)	Reduce waiting times: immediate or same-day results, being mindful of delays from holidays, results over phone or email Avoid unnecessary scans Defer scans until after birthdays or important events Discuss preferences of scans and result delivery with patients Assist patient preparedness for scan results by pre-emptively discussing future treatment options Providing compassionate care
	Clinician actions (radiology staff)	Being mindful of language used in scan reports Have experienced staff perform intravenous cannulation Being mindful of music during a scan (eg, do not play depressing music)
	Health care delivery	Direct patient access to scan results Providing assistance to patients around navigation of health care systems Improved insurance pathways when approval for scans is needed Providing contact details for medical or nursing staff for questions

General strategies included distraction, physical and mental methods of relaxation, spiritual practices, and seeking support or professional help. Specific strategies included adopting a helpful psychological approach and using practical strategies. Users gained some control over their situations by reducing the time until they received results, taking antianxiolytics, or building relationships with their health care team. Strategies that required involvement by health care professionals or systems included patient and clinician education, actions by oncologists or radiology staff, and considerations around health care delivery.

#### Theme 5: Research About Scanxiety

This theme included publications, conference presentations, or news discussing research. The research included the prevalence and severity of scanxiety, preferences for expedient results, and the impact of scanxiety on families. Research about fear of recurrence, frequency of scans, and cost-benefit ratios in cancer surveillance was tied back to scanxiety. The research described ways to reduce scanxiety through medical hypnosis, educational patient videos, the use of miniature magnetic resonance imaging (MRIs) scans, Lego MRIs, or open MRIs, the use of virtual reality, and the alternate use of tumor markers. Tweets about research were mostly posted by organizations, researchers, and clinicians.

## Discussion

### Principal Findings

This observational study explored activity on Twitter about scanxiety over the 3-year study period. Conversations about scanxiety were most commonly initiated by women with breast cancer. There were 3623 conversations about scanxiety, averaging 101 conversations per month. Most tweets used the term “scanxiety.” Users often shared their personal experiences about scanxiety (60% of conversations), with one-fifth of primary tweets containing hyperlinks, media, or emojis.

The need to recognize and manage scanxiety was evident. Users shared and labeled experiences as scanxiety when describing their own situations, supporting others, or providing commentary on the research, increasing awareness and acceptance of this term. The relatability of scanxiety appeared to unify members of cancer communities across a range of cancer types, despite diverse descriptions of their experiences. The importance of scanxiety was reflected in the number of organizations initiating scanxiety conversations (n=254), with these users potentially reaching a broader readership than individuals. Further, as increasing cancer incidence and improved cancer survival leads to an increased number of scans for patients, there is likely to be a corresponding increase in the relevance, applicability, and impact of scanxiety.

There are discrepancies between existing research on scanxiety and the priorities that emerged from our work. Existing observational research has focused on the physical aspects of scans [9-12,14]. This was also seen in the scoping review on scanxiety, where all 10 intervention studies to reduce scanxiety focused on the scan itself [8]. In contrast, conversations about scanxiety by Twitter users often related to the presence, duration, and degree of uncertainty arising from scans and scan results, mirroring research in people with cancer where uncertainty about cancer trajectory and prognosis increases psychosocial worries from fear of cancer recurrence or progression [2,21]. Interventions proposed by Twitter users to reduce scanxiety involved systemic changes centered around health care delivery, such as improved processes around scan reporting times, digital access to results, and patient education about scan procedures and scanxiety. Users also advocated for improved patient navigation services to assist with timely scan bookings and results and ensure open communication between clinicians and patients. Notably, some strategies described or proposed by Twitter users, such as being mindful of the timing of scans in relation to appointments or birthdays, could be adapted into standard clinical practice without substantial cost or resource use.

### Strengths and Limitations

The strengths of this study include capturing a multicountry perspective on a relatable problem across cancer types and stages and using a novel, resource-considerate approach to data collection using a thorough and systematic search strategy. Data collection from social media platforms can allow the capture of real-time experiences from a diverse range of people who may not otherwise participate in research, providing supplementary data to traditional methodologies.

The primary limitation of this study was an unavoidable selection and reporting bias. Patient demographics in our study do not match global cancer statistics, with disparate proportions observed in sex, age, and cancer type compared with either global or North American populations [22,23]. Compared to the general population, Twitter users are also more likely to be more educated, have higher incomes, and have higher digital literacy [24]. Experiences related to scanxiety could be under- or overrepresented by users who were comfortable publicly sharing their experiences, with additional bias introduced through the inclusion of only English-language tweets and the exclusion of unavailable tweets due to user removal, privacy settings, or deleted user accounts. Further, our included search terms may not have captured all tweets about scanxiety, as different words or phrases may be used by other users to describe this experience. Data available in tweets and on user profiles is also subject to reporting bias, as this data cannot be verified. Given the significant selection and reporting bias, we did not attempt to quantify the prevalence or severity of scanxiety from our data. This research should be used to supplement data collected using other methodologies rather than as a stand-alone information resource.

Other limitations include the manual search, data extraction, and analysis of Twitter data, which are less efficient and more susceptible to human error than automated processes. We were restricted by a lack of resources, though we note that research using social media is a new arena for data collection and analysis. Automated processes are being developed and could be used effectively in future studies. For example, since our data collection concluded, Twitter has upgraded its application programming interface to improve access to publicly available data on Twitter for research [25].

### Conclusions

Scanxiety is experienced individually by people having cancer-related scans and is likely to increase in significance as the number of people living with cancer and having cancer-related scans increases over time. This research provides clinicians with a starting point to understand and improve scanxiety. It demonstrates how social media platforms can be used to explore psychosocial health issues in the cancer community, though researchers must allow for bias when interpreting results.

Acknowledging scanxiety as a term and as a “real,” lived experience for people with cancer will improve awareness of how clinicians explain, order, and organize scans and scan results. This study identified low-cost and low-resource practical strategies to reduce scanxiety that could be rapidly introduced into clinical care.

Further scanxiety research priorities include understanding the longitudinal trajectory of scanxiety around and between scans and determining an evidence-based approach to reduce scanxiety. Given the potential breadth of scanxiety across all people having cancer-related scans, this likely requires system-based changes.

## Appendix

Multimedia Appendix 1 Search term included in Twitter conversations about scanxiety.

Multimedia Appendix 2 Categories and examples of links, media and emojis.

Multimedia Appendix 3 A compilation of words and phrases used to describe scanxiety, as extracted from tweets. Greater text size reflected the manual selection and weighting of words and phrases with greater emotional impact, as well as automatic adjustments made by the inbuilt algorithms of the word art creator.

Multimedia Appendix 4 Expressions of support – examples .

## Abbreviations

MRI: magnetic resonance imaging

## Data Availability

The data sets generated during this study are available at reasonable request from the corresponding author. This includes the complete list of deidentified tweets and demographic information.
