# Supplementary material for: Scanxiety Conversations on Twitter: Observational Study
Source: JMIR Cancer. 2023 Apr 19;9:e43609. doi: 10.2196/43609 (PMC10157462; doi:10.2196/43609)
Supplement: Multimedia Appendix 1 [file cancer_v9i1e43609_app1.docx]

| Search term used | n (%) |
| --- | --- |
|  |  |
| Scanxiety | 3312 (91) |
| Scan anxiety | 125 (3) |
| Scananxiety | 68 (2) |
| Other, including scan-anxiety, scan-associated anxiety, scan-associated distress, scan-related anxiety, scanziety or multiple terms used | 25 (1) |
| Multiple search terms included | 3 (<1) |
| No search term included | 153 (4) |
