# Supplementary material for: Scanxiety Conversations on Twitter: Observational Study
Source: JMIR Cancer. 2023 Apr 19;9:e43609. doi: 10.2196/43609 (PMC10157462; doi:10.2196/43609)
Supplement: Multimedia Appendix 2 [file cancer_v9i1e43609_app2.docx]

| Category |  | *n* (%) | Examples |
| --- | --- | --- | --- |
|  |  |  |  |
| **Links** |  |  |  |
|  | Blog | 414 (55) | Social media (e.g., Instagram, Facebook) or dedicated blogging platform (e.g., WordPress, BlogSpot) of the Twitter user |
|  | Strategies to reduce scanxiety | 153 (21) | Webpages hosted by individuals or cancer organisations about strategies or tips and tricks to reduce scanxiety |
|  | News articles | 139 (19) | Articles published as part of a bigger organisation than the individual Twitter user, including news organisations (New York Times, the Guardian, Digital Health Buzz) or cancer organisations (e.g., Cure, The Big Ordeal), on topics not covered by the other categories |
|  | Research | 10 (1) | Publications or news articles about research |
|  | Other | 30 (4) | Advertisements to events |
| **Media** |  |  |  |
|  | Twitter user | 206 (29) | Themselves, including self-taken photos |
|  | Humour | 91 (13) | Quotes, memes or video snippets encouraging laughter |
|  | Scan | 90 (13) | Scan machines, signs for radiology or cannulas inserted in a user’s arm |
|  | Inspirational text | 71 (10) | Quotes or images instilling hope or serenity |
|  | Definition of scanxiety | 68 (10) | Media containing text that defines scanxiety |
|  | Advertisement | 62 (9) | Promotion of webinars or support groups |
|  | Medical | 26 (4) | Scan reports or images |
|  | Strategies to reduce scanxiety | 35 (5) | Media containing text that recommends strategies |
|  | Research | 35 (5) | Conference presentations |
|  | Other | 25 (4) | Media containing text of a quote, news headline or cup of coffee |
| **Emojis** |  |  |  |
|  | Positive | NA | 🤗😊😄🙃😁 |
|  | Negative | NA | 😟😞😭🤢😬 |
|  | Supportive | NA | 🙏🏻👌💪✌🏼🙌🏾 |

NA Not available (this data was not recorded by the research team)
