# Supplementary figures and images for: Scanxiety Conversations on Twitter: Observational Study
Source: JMIR Cancer. 2023 Apr 19;9:e43609. doi: 10.2196/43609 (PMC10157462; doi:10.2196/43609)

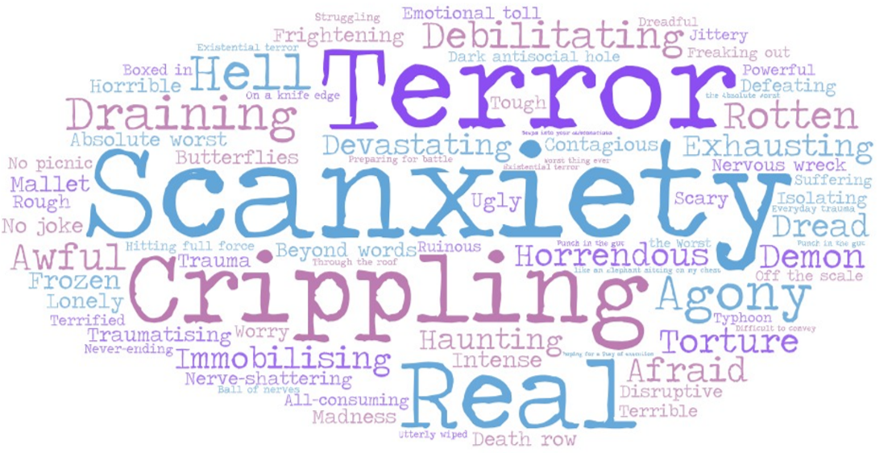

Supplement: Multimedia Appendix 3 [file cancer_v9i1e43609_app3.png]
