# Supplementary material for: Scanxiety Conversations on Twitter: Observational Study
Source: JMIR Cancer. 2023 Apr 19;9:e43609. doi: 10.2196/43609 (PMC10157462; doi:10.2196/43609)
Supplement: Multimedia Appendix 4 [file cancer_v9i1e43609_app4.docx]

| Category | Examples |
| --- | --- |
|  |  |
| Well wishes | ‘Fingers crossed’; ‘Good luck’; ‘Praying of you; ‘Sending love’ |
| Encouraging positivity | ‘Hang in there’; ‘Keep smiling’ |
| Reassurance:  Scanxiety is normal | ‘Scanxiety is perfectly normal’;  ‘What you’re feeling and thinking is normal’ |
| Reassurance:  Scanxiety is relatable | ‘Only your tribe can fully understand’;  ‘I know exactly how you feel, I’ve been there’ |
| Reassurance – support from others | ‘Know you are not alone’;  ‘Whatever I can do to help’ |
